# Adult-Onset Cervical Lymphatic Malformation: A Case Report

**DOI:** 10.7759/cureus.93564

**Published:** 2025-09-30

**Authors:** Rayan Zarei, Haralambos Andrianakos, Kathryn Vidlock

**Affiliations:** 1 Department of Biomedical Sciences, Rocky Vista University College of Osteopathic Medicine, Parker, USA; 2 Department of Family Medicine and Ultrasound, Rocky Vista University College of Osteopathic Medicine, Parker, USA

**Keywords:** adult neck mass, alpelisib, lymphatic malformation, parotid tail lesion, sclerotherapy, sirolimus, vascular malformation

## Abstract

Lymphatic malformations (LMs) are low-flow vascular malformations of lymphatic origin that are typically diagnosed in childhood, with cervicofacial LMs posing particular risks for functional and cosmetic morbidity. Fewer than 10% of LMs present after adolescence, and adult-onset cervical cases are exceedingly rare, with only isolated reports in the literature. Adult-onset cervical LMs often mimic other cystic neck lesions, complicating diagnosis and delaying management.

We report the case of a 20-year-old male who presented with a painless, slowly enlarging left upper neck mass. Ultrasound demonstrated a 3.2 × 3.0 × 1.4 cm multiloculated cystic avascular lesion, while contrast-enhanced CT revealed a 6.7 × 2.7 × 2.2 cm multiloculated cystic mass located between the parotid tail and the sternocleidomastoid muscle. MRI confirmed a T2-hyperintense, multiseptated lesion with areas of macroscopic intralesional fat and minimal enhancement, findings most consistent with LM rather than teratoma. Follow-up MRI nine months later demonstrated stability (2.7 × 1.2 × 3.7 cm).

Active surveillance was chosen over intervention due to the patient’s asymptomatic status, stable morphology, and the potential morbidity associated with surgical or sclerotherapeutic management. Adult cervical LMs remain diagnostically challenging due to overlap with branchial cleft cysts, ranulas, venous malformations, and cystic nodal metastases. The presence of intralesional fat can further complicate diagnosis, as it is more typically associated with dermoid cysts or teratomas, yet has been documented in LMs.

Multimodal imaging is essential, with MRI offering the greatest sensitivity for delineating lesion extent. Management should be individualized: surveillance is appropriate in stable, asymptomatic cases, whereas progressive or symptomatic lesions may require sclerotherapy, surgical excision, or systemic therapies such as sirolimus or PI3K inhibitors. This case underscores the importance of including LM in the differential diagnosis of adult cervical cystic lesions and highlights that conservative management is often safe in clinically stable, asymptomatic patients.

## Introduction

Lymphatic malformations (LMs) are congenital, low-flow vascular malformations caused by abnormal embryologic lymphatic development [1]. They are generally identified in early childhood, with 80-90% detected before the age of two [2]. Cervicofacial involvement is common due to the embryologic predominance of lymphatic structures in this region [3].

While LMs are typically pediatric diagnoses, adult-onset cervical presentations are exceedingly uncommon. Fewer than 10% of cases present after adolescence, and the literature on adult incidence is limited to isolated case reports and small series [4,5]. In adults, cervical cystic masses are more often attributed to branchial cleft cysts, ranulas, or metastatic cystic lymph nodes, complicating diagnosis [6]. Radiologic features are central to differentiation: for example, LMs typically appear as multiloculated, T2-hyperintense lesions without significant enhancement, whereas branchial cleft cysts are usually unilocular, dermoids often contain fat, and nodal metastases may show solid enhancing components. MRI is considered the gold standard for diagnosing LMs due to its superior ability to delineate lesion extent, while histopathologic examination may be required for definitive diagnosis in atypical or ambiguous cases [5,7]. Correct identification is essential, as management ranges from conservative surveillance to invasive interventions such as sclerotherapy, surgical excision, or systemic pharmacotherapy [8,9].

This case is notable for three reasons: the lesion presented in adulthood, its location was at the parotid tail adjacent to the sternocleidomastoid muscle, and the presence of macroscopic intralesional fat was observed. This latter feature is unusual, with only isolated cases reported in the literature, and is diagnostically significant because it can mimic dermoid cysts or teratomas [10]. We present this case to highlight an uncommon adult-onset cervical LM with intralesional fat, emphasize the diagnostic challenges of fat-containing LMs, and illustrate that active surveillance can be an appropriate management strategy in asymptomatic, stable cases.

## Case presentation

A 20-year-old male with no significant past medical history presented with a painless, slowly enlarging swelling in the left upper neck, first noted approximately three months prior to presentation. The lesion had shown gradual progression without acute changes. The patient denied dysphagia, dyspnea, odynophagia, fever, or systemic symptoms. He had no history of trauma, prior surgery, or radiation.

On physical examination, there was a soft, mobile, compressible, and non-tender mass located along the posterior margin of the left parotid tail, anterior to the sternocleidomastoid muscle. No overlying skin changes, pulsatility, bruit, or cervical lymphadenopathy were identified. Cranial nerve function was intact. The Valsalva maneuver was not performed during the examination. Baseline laboratory evaluation, including complete blood count and metabolic panel, was unremarkable, with no evidence of infection or systemic disease.

**Figure 1:**
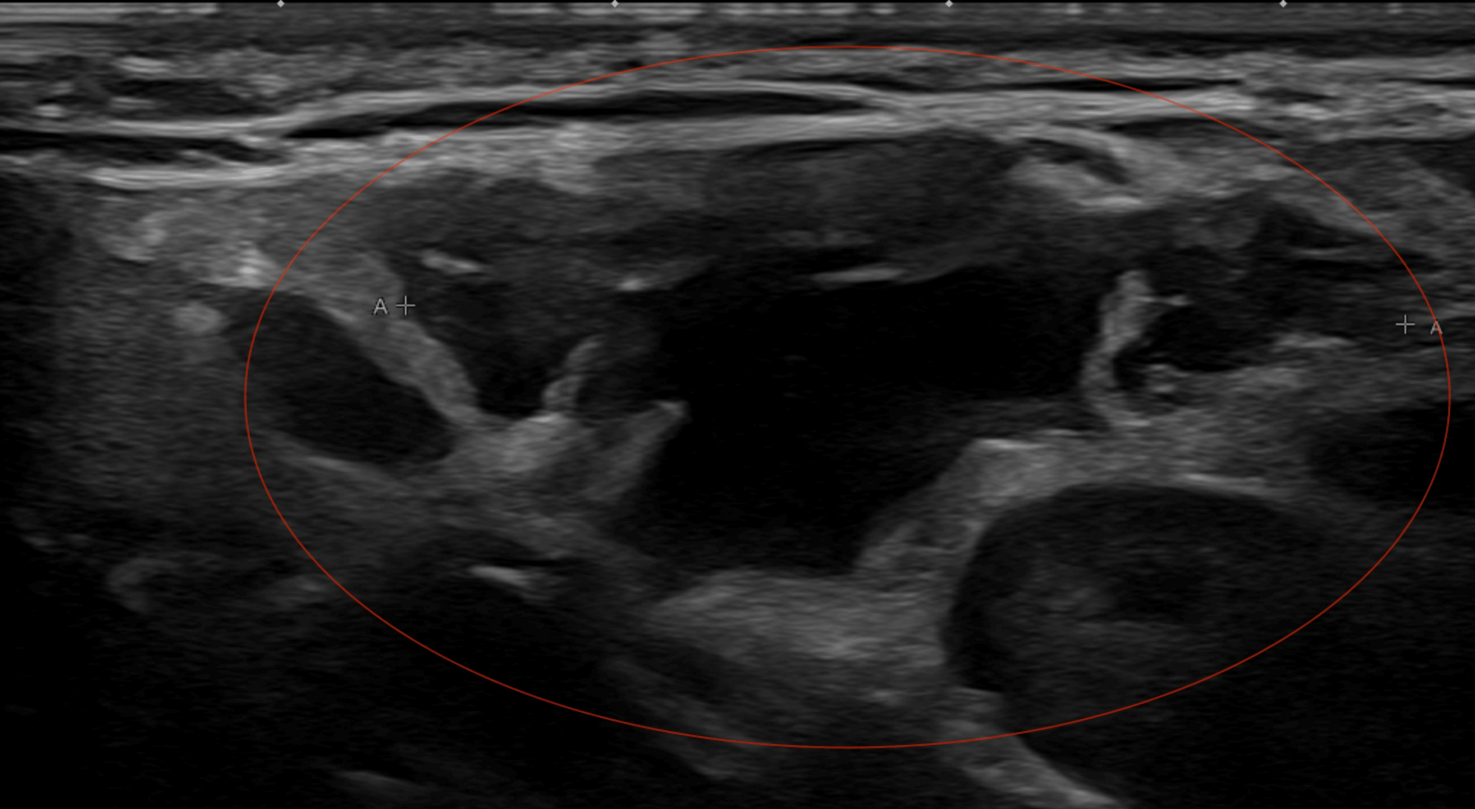
Ultrasound of the left upper neck demonstrating a multiloculated, avascular cystic lesion (circled in red) located between the parotid tail and the sternocleidomastoid muscle.

**Figure 2:**
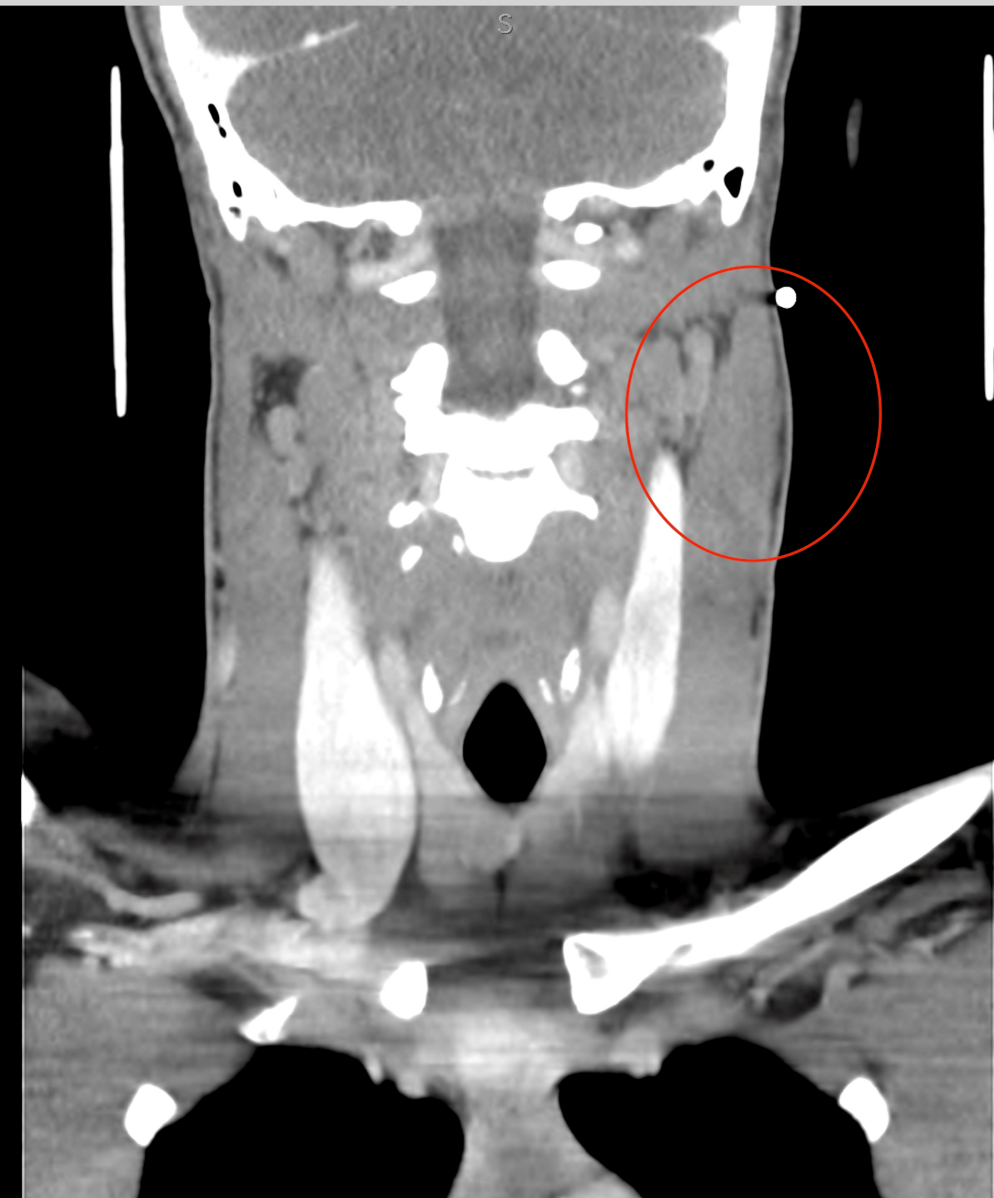
Contrast-enhanced CT showing a multiloculated cystic lesion (circled in red) between the left parotid tail and the sternocleidomastoid muscle.

**Figure 3:**
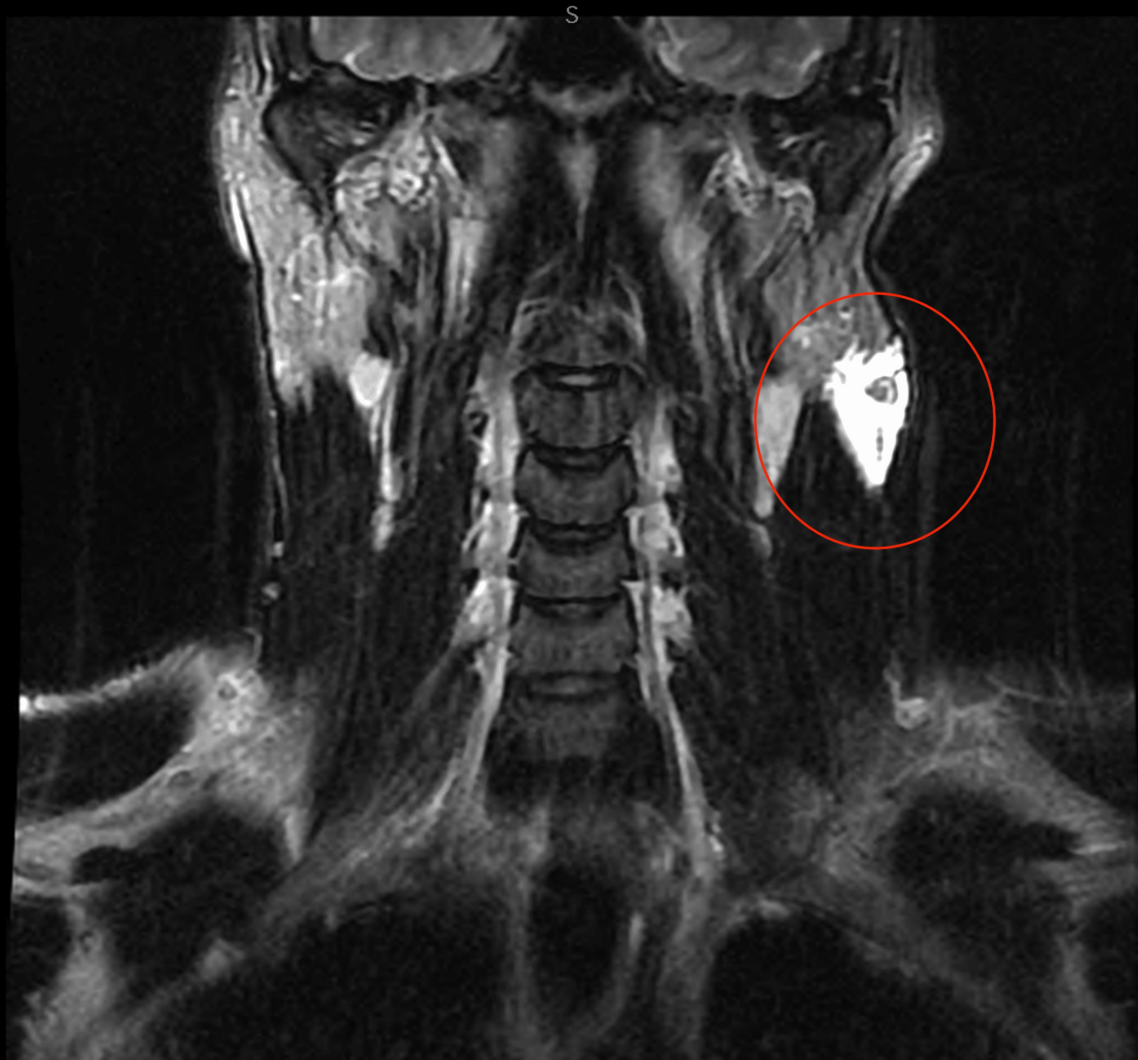
Axial T2-weighted MRI showing a multiseptated, hyperintense lesion (circled in red) with intralesional fat and minimal enhancement.

**Figure 4:**
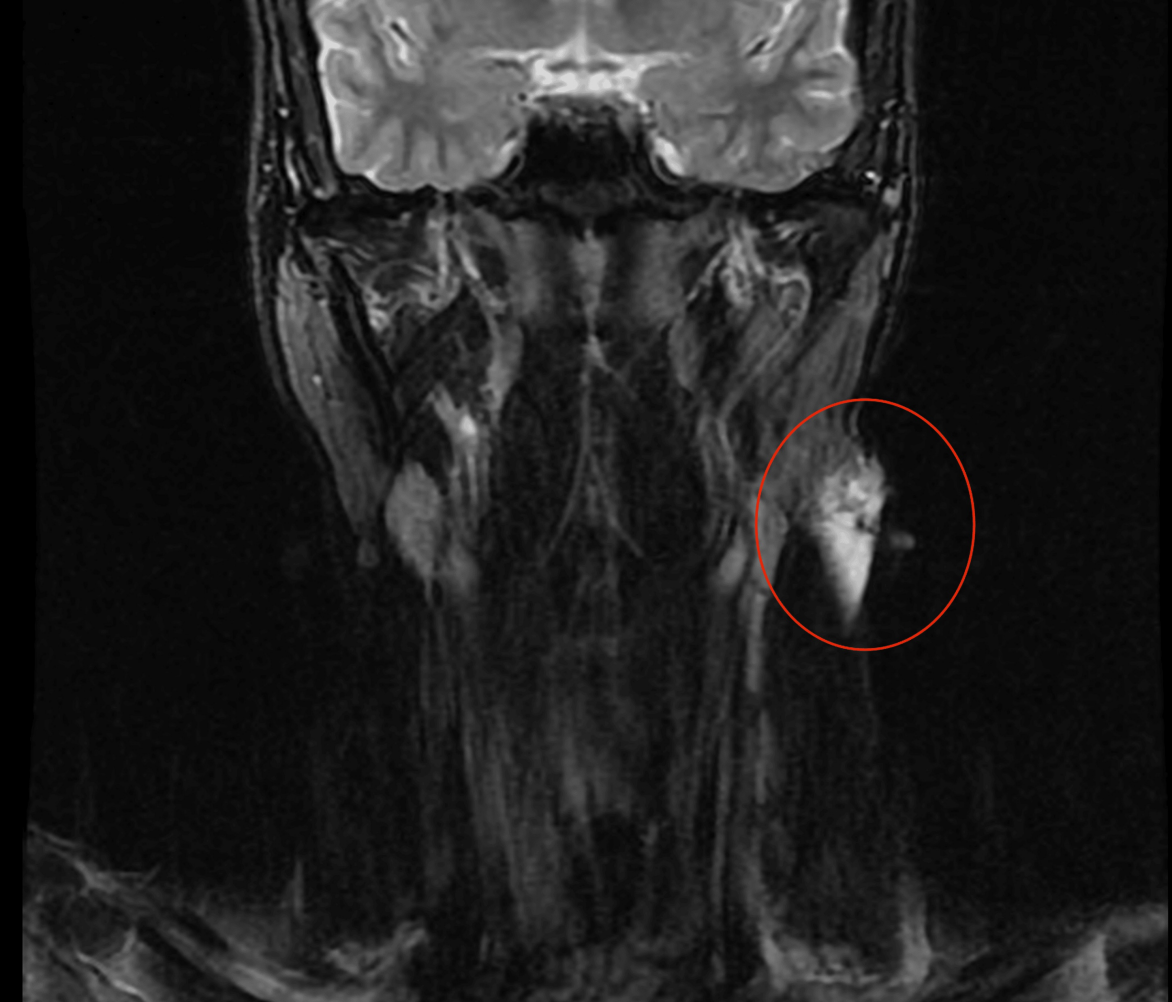
Coronal T2-weighted MRI showing stability in lesion size and morphology over time (lesion circled in red).

Ultrasound was obtained as the initial modality for characterization and demonstrated a 3.2 × 3.0 × 1.4 cm multiloculated avascular cystic lesion in the left anterior neck (Figure 1). Contrast-enhanced CT of the neck was then performed on July 27, 2022, to further assess lesion extent and exclude vascular or infiltrative features. It revealed a 6.7 × 2.7 × 2.2 cm multiloculated cystic lesion located between the left parotid tail and the sternocleidomastoid muscle, without sublingual extension or abnormal enhancement (Figure 2). MRI performed on August 22, 2022, provided superior tissue characterization and showed a multiseptated, T2-hyperintense lesion with areas of intralesional macroscopic fat, an atypical feature that significantly narrowed the differential diagnosis, and minimal enhancement, findings that favored LM over cystic teratoma (Figure 3). A follow-up MRI on April 20, 2023, demonstrated stability of the lesion, measuring approximately 2.7 × 1.2 × 3.7 cm (Figure 4).

Given the absence of symptoms, preserved airway and swallowing function, and stability across imaging modalities, the decision was made for active surveillance with regular clinical follow-up and interval imaging. The patient was counseled on warning symptoms, including rapid enlargement, pain, infection, or airway compromise. At the most recent follow-up in April 2023, the lesion remained stable with no clinical changes.

This case report was conducted in accordance with the principles of the Declaration of Helsinki. Institutional ethical approval was obtained from Rocky Vista University, College of Osteopathic Medicine, with protocol ID 2025-122, approved on 04/18/2025. Written informed consent was obtained from the patient for participation and publication of anonymized clinical details and images.

## Discussion

Lymphatic malformations (LMs) represent developmental anomalies rather than true neoplasms, arising from isolated lymphatic sacs that fail to establish communication with the central lymphatic system [1]. While the majority are identified in childhood, a subset of patients may present in adulthood, either incidentally or due to new symptoms. Enlargement in adults is typically triggered by superimposed infection, hemorrhage, or trauma [4,6].

In adults, the differential diagnosis of a cystic cervical lesion is broad and includes LM, branchial cleft cyst, plunging ranula, dermoid cyst or teratoma, venous malformation, and cystic nodal metastasis. Radiologic features help narrow this differential. LMs classically appear as multiloculated, thin-walled cystic lesions with high T2 signal intensity and minimal enhancement [2,6]. Branchial cleft cysts usually localize near the mandibular angle and may exhibit the characteristic “notch sign” [5], whereas ranulas arise in the sublingual space and demonstrate a “tail sign” [6]. Dermoids and teratomas are distinguished by the presence of fat or calcifications, generally in a midline location [7]. Venous malformations often contain phleboliths with contrast enhancement [7], while cystic nodal metastases must be strongly considered in patients with a history of mucosal malignancy [11].

Each imaging modality contributes unique diagnostic value. Ultrasound is an accessible first-line tool that demonstrates cystic composition and avascularity, though it is subject to operator variability [12]. CT is particularly useful for delineating lesion compartments and detecting fluid-fluid levels in the setting of hemorrhage [13]. MRI provides the greatest sensitivity for disease characterization and extent, and is particularly valuable for distinguishing benign congenital lesions such as LMs from malignant metastatic lymphadenopathy [5,13]. Recent reviews emphasize that cystic nodal metastases, particularly from human papillomavirus-associated oropharyngeal carcinoma, can closely mimic benign cystic lesions such as LMs, branchial cleft cysts, or venous malformations. Features that favor metastasis include the presence of internal or irregular enhancing soft tissue, thick septations, and restricted diffusion, with correlation to evaluation for a primary mucosal tumor recommended [11,13]. Rarely, as in the present case, intralesional fat may be identified. While this finding can mimic teratoma or dermoid, prior reports confirm that fat can occur within LMs. This atypical feature is clinically important, as it can create diagnostic uncertainty and, if unrecognized, may lead to unnecessary invasive procedures [10].

Minor discrepancies in lesion size across imaging modalities are not uncommon, as they may reflect differences in slice thickness, technique, or measurement orientation. Interobserver variability of approximately 5-10% has been reported for such lesions [12,14]. Accordingly, interval stability across serial imaging studies carries more clinical significance than small numerical differences in measurement.

Management decisions for LMs are guided by symptom burden, lesion growth, and anatomical considerations. Observation is reasonable in asymptomatic, stable cases such as this one [6]. Intervention is generally indicated in cases of progressive enlargement, recurrent infection, pain, cosmetic or functional impairment, or when lesions compromise vital structures such as the airway or swallowing mechanism [6,15]. Interventional options are reserved for progressive or symptomatic disease. Sclerotherapy is the preferred treatment for macrocystic lesions and can be performed with agents including bleomycin, doxycycline, or OK-432 [15]. Surgical excision remains an option for refractory cases but carries the inherent risk of damage to adjacent neurovascular structures [3,15]. Current multidisciplinary consensus statements emphasize that management decisions should ideally be made within a team setting, including otolaryngology, interventional radiology, radiology, dermatology, and genetics, guided by published pathways that recommend individualized treatment sequencing based on lesion growth, function, and cosmesis [16,17]. Long-term outcomes vary by treatment modality; recurrence is common after incomplete excision or sclerotherapy, and complications may include infection, scarring, or neurovascular injury [3,15]. These advances reflect the growing recognition that many LMs are driven by somatic mutations in the PIK3CA-AKT-mTOR signaling pathway, which provides the biologic rationale for targeted therapies. More recently, targeted systemic therapies have broadened treatment strategies for unresectable or diffuse disease. Sirolimus, an mTOR inhibitor, and alpelisib, a PI3K inhibitor, have demonstrated efficacy in reducing lesion burden and improving quality of life in patients with complex LMs [9,18].

This case highlights the need to maintain LM in the differential diagnosis of adult cervical cystic lesions, particularly when atypical features such as intralesional fat are present. Recognizing these unusual imaging findings is critical to avoid misdiagnosis, prevent unnecessary invasive procedures, and guide appropriate management. This report is limited by the absence of histopathologic confirmation, as the diagnosis was based on characteristic imaging features and clinical stability. Genetic testing was not performed, and follow-up was limited to one year, which constrains conclusions regarding long-term progression or treatment response.

## Conclusions

Adult cervical LMs are rare but clinically significant. Early and accurate diagnosis is essential to prevent mismanagement, particularly given their overlap with congenital and malignant cystic neck lesions. Multimodal imaging is critical for distinguishing these lesions, and recognition of atypical features such as intralesional fat can directly influence decision-making by helping avoid unnecessary invasive procedures. Conservative observation with close follow-up is appropriate for stable, asymptomatic cases, whereas individualized treatment, guided by lesion type, location, and patient-specific factors, may involve sclerotherapy, surgery, or systemic therapy in progressive or symptomatic disease.
